# Spindle-Integrated Three-Axis Cutting Force Measurement System for Ultra-Precision Diamond Milling

**DOI:** 10.3390/s26061817

**Published:** 2026-03-13

**Authors:** Zhongwei Li, Liang An, Yuqi Ding, Huanbin Lin, Yuan-Liu Chen

**Affiliations:** The State Key Lab of Fluid Power and Mechatronic Systems, Zhejiang University, Hangzhou 310027, China; liang_an@zju.edu.cn (L.A.); dingyuqi8@zju.edu.cn (Y.D.); huanbinlin@zju.edu.cn (H.L.)

**Keywords:** cutting force measurement, ultra-precision diamond milling, piezoelectric force sensor

## Abstract

Ultra-precision diamond milling is a crucial technology for machining precision components with complex-shaped surfaces and microstructure array surfaces. Machining process monitoring is a promising approach to improving machining quality. This paper proposes a spindle-integrated three-axis cutting force measurement method for ultra-precision diamond milling using force piezoelectric force sensors. A spindle-integrated force measurement mechanism utilizing four piezoelectric force sensors arranged symmetrically and diagonally for measuring three-axis cutting forces was designed. Calibration tests showed that the linearity of force detection in three directions was less than 2%. Tool-setting experiments based on force detection signals were conducted, demonstrating the capacity of precision tool-setting in the Z-direction with an accuracy of less than 100 nm. A Wiener filter was employed to eliminate measurement noise from vibration and inertial forces under spindle rotation. Ultra-precision milling experiments were carried out based on the designed spindle-integrated force measurement mechanism, and the measurement results demonstrated that the system could effectively detect cutting forces below 50 mN and exhibited good correlation with the measurement results of commercial standard dynamometers. This paper provides a promising and effective in-process force measurement technology for the ultra-precision milling process.

## 1. Introduction

Ultra-precision diamond milling is a highly efficient machining technology for fabricating complex-shaped surfaces and microstructure array surfaces [1,2]. Monitoring the milling process is an important method for providing feedback of the machining status and further improving machining quality [3,4]. Since cutting force is a significant factor of the material removal process of milling, in-process cutting force measurement serves as an effective approach to realizing milling process status monitoring [5,6]. With the increasingly growing requirements for machining accuracy of precision components, milling force measurement is expected to possess high-sensitivity down to the millinewton-level and multi-dimensional force measurement capability [7].

Cutting forces can be indirectly calculated from relevant parameters such as the spindle current of the machine tool; yet this method suffers from the drawbacks of low sensitivity and narrow bandwidth [8,9]. The use of external force sensors for measurement is an effective approach to improving measurement sensitivity and bandwidth [10,11]. Commercial platform dynamometers are commonly adopted for milling force measurement, which require workpieces to be mounted on the dynamometers [12,13]. However, due to the limited mounting dimensions of such dynamometers, it is difficult to install large-size workpieces.

To address this problem, integrating the force measurement module into the spindle end is an effective solution. Force measurement rotational tool holders used in milling processes have been studied in recent years, mainly based on capacitive sensors [14,15,16], strain gauges [17,18,19], and piezoelectric sensors [11,20,21]. Xie et al. designed a four-component force measurement tool holder based on capacitive sensors [14]. Qin et al. developed a tool holder for measuring axial force and torque using high-sensitivity semiconductor strain gauges [18]. Yau et al. developed a sensory tool holder by inserting piezoelectric force sensors into a standard tool holder [21]. However, milling spindles of ultra-precision machine tools can achieve high rotational speeds of tens of thousands of revolutions per minute, while the tools mounted on these spindles are relatively light in weight. For this reason, the force measurement rotational tool holders mentioned above are not suitable for ultra-precision milling applications owing to their relatively large mass.

Therefore, to address this, numerous spindle-integrated force measurement methods that do not add rotational mass or alter the original structure of the spindle have been investigated. Andreas et al. measured radial cutting force indirectly by using a high-resolution displacement sensor to detect the force-induced deformation at the front end of the spindle; however, the high system stiffness resulted in low sensitivity, with a measurement resolution of approximately 1.5 N [22]. Kim et al. and Sarhan et al. adopted a similar approach, employing four symmetrically arranged displacement sensors to achieve two-dimensional milling force measurement [23,24]. Denkena et al. machined symmetric notches on the spindle to enhance local strain and mounted metal strain gauges at the notch locations for three-dimensional force measurement [25]. Boujnah et al. utilized four high-precision semiconductor strain gauges symmetrically mounted on the spindle to realize three-dimensional force measurement [26]. Nevertheless, the force measurement methods mentioned above exhibit relatively low sensitivity, being only capable of resolving forces at the Newton level or above, which renders them unsuitable for ultra-precision milling processes. In contrast, piezoelectric force sensors feature higher force measurement resolution and have been successfully applied in ultra-precision turning processes by the authors [27,28,29]. However, to date, there are relatively few studies on the application of piezoelectric force sensors in ultra-precision milling processes.

This paper proposes a novel spindle-integrated three-axis cutting force measurement method for ultra-precision diamond milling using force piezoelectric force sensors. A spindle-integrated force measurement mechanism utilizing four piezoelectric force sensors arranged symmetrically and diagonally to measure three-axis cutting forces was designed. Calibration tests and tool-setting experiments were carried out to verify the static performance of the system. Ultra-precision milling experiments were conducted, and the results exhibited good correlation with the measurement results of a commercial standard dynamometer, thus verifying the in-process dynamic force measurement capability of the proposed method for ultra-precision milling processes.

## 2. Structure Design

**Figure 1:**
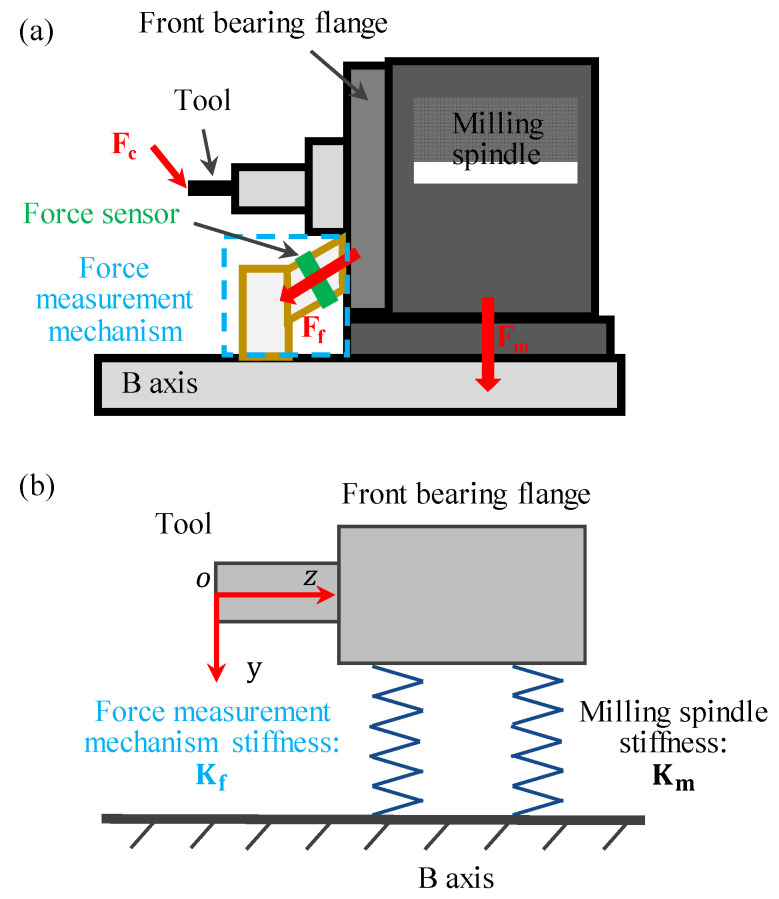
(**a**) Mechanical schematic diagram of the milling axis and force measurement mechanism; (**b**) schematic of stiffness.

This paper intends to establish a force measurement mechanism on a five-axis ultra-precision machine tool (Moore Nanotech 650FG, USA). Figure 1 shows the mechanical schematic diagram. The milling spindle is mounted on the B-axis. Since a large proportion of the cutting forces generated during the milling process are transmitted through the front bearing flange of the spindle, the front bearing flange is identified as a potential location for mounting force sensors. Considering the ample space available on the front bearing flange of the milling spindle and the surface of the B-axis, the force measurement mechanism was designed that one side is connected to the front bearing flange and the other side is connected to the B-axis, as shown in Figure 1a. The force measurement mechanism consists of piezoelectric force sensors and a force transmission structure. The piezoelectric sensors were arranged with a deflection angle relative to all three axial directions for sensitive measurement of both axially and radially applied forces. The magnitude of the force flux passing through the force measurement mechanism is determined by its stiffness, which determines the sensitivity of force measurement. The relationship among the cutting forces acting on the milling cutter **F_c_**, the force component transmitted through the force measurement mechanism **F_f_**, and the force component borne by the milling spindle **F_m_** is as follows:

**F_c_** = **F_f_** + **F_m_** (1)

Figure 1b shows the force transmission model, and the magnitude of the force flux passing through the force measurement mechanism can be simplified as:

(2) $$F_{f}=\frac{F_{c}K_{s}}{{K_{s}+K}_{m}}$$

where **K_s_** and **K_m_** are the stiffnesses of the force measurement mechanism and the milling spindle. It should be noted that the spindle stiffness was assumed to be constant, and the influence of the dynamic milling process on spindle stiffness can be ignored due to the small cutting force of less than several Newtons in the ultra-precision milling process.

**Figure 2:**
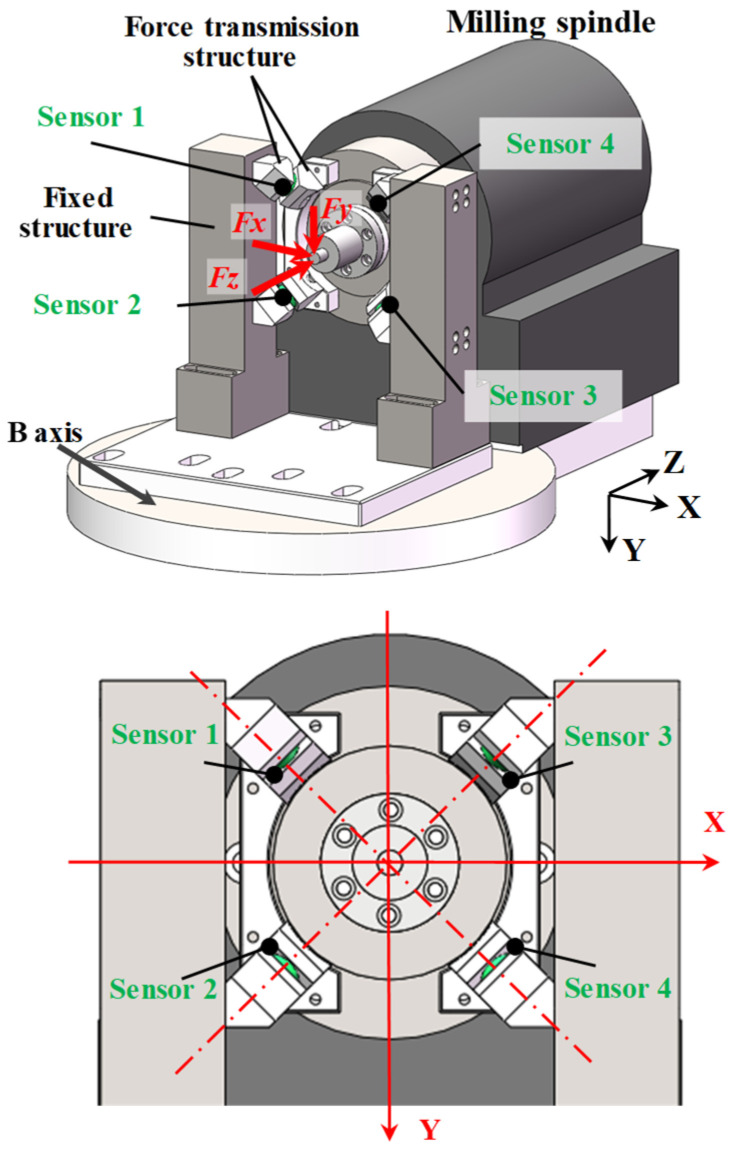
Structure of the force measurement mechanism.

Figure 2 shows the structure of the force measurement mechanism. Four piezoelectric sensors were utilized for simultaneous measurement of three-axis cutting forces. To minimize the crosstalk between the measured signals, the four piezoelectric sensors were designed to be aligned along their respective diagonal directions and mounted at symmetrical positions. The sensors were aligned with a deflection angle of 45° along the XY axes for receiving forces from three axial directions. Based on this configuration, the relationship between the three-axis cutting forces and the sensors’ outputs can be expressed as follows:

(3) $$[F_{x}\;F_{y}\;F_{z}{]}^{T}=K\;\cdot \;[U_{1}\;U_{2}\;U_{3}\;U_{4}{]}^{T}$$

where *U*_1_, *U*_2_, *U*_3_, and *U*_4_ are the voltage outputs of the four sensors. **K** is a transformation matrix from the outputs of the sensors to the three-axis forces, which is related to factors such as the preload of the force sensors, the symmetry of mounting positions, and the stiffness of the force transmission structures, and will be obtained by calibration tests.

## 3. Experiment Setup and Test

**Figure 3:**
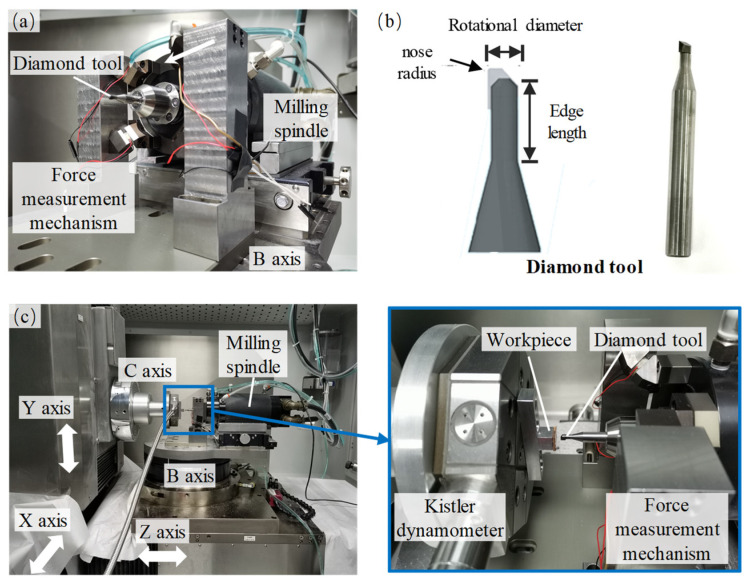
Photograph of the experimental setup: (**a**) force measurement mechanism; (**b**) diamond tool; (**c**) setup of test and cutting experiments.

Figure 3 shows a photograph of the designed force measurement mechanism and the experimental setup on an ultra-precision milling machine tool. As shown in Figure 3a, the force measurement mechanism was mounted between the B-axis and the milling spindle of the machine tool. The force sensor adopted is a ring-shaped piezoelectric ceramic (Coremorrow NAC2123, China). The outputs of the sensors were collected by a homemade charge amplifier. To ensure the high-stiffness of the force measurement mechanism, all components of the designed device were made of alloy steel. Figure 3b shows a photograph of the used diamond milling tool, with a nose radius of 0.1 mm, an edge length of 6 mm, a rotational diameter of 4 mm, and a shank diameter of 6 mm. The diamond milling tool was fixed on the spindle by a clamp with an inner hole diameter of 6 mm. As shown in Figure 3c, a standard dynamometer (Kistler 9109AA, Switzerland) was installed on the C-axis of the machine tool. The diamond tool was mounted on the milling spindle via a collet, and the workpiece was fixed onto the standard dynamometer. The machine tool employed features a five-axis configuration, where the X-axis and Y-axis can drive the workpiece to move along the X- and Y-directions, respectively, and the Z-axis can drive the milling spindle to move along the Z-direction.

### 3.1. Homemade Charge Amplifier

**Figure 4:**
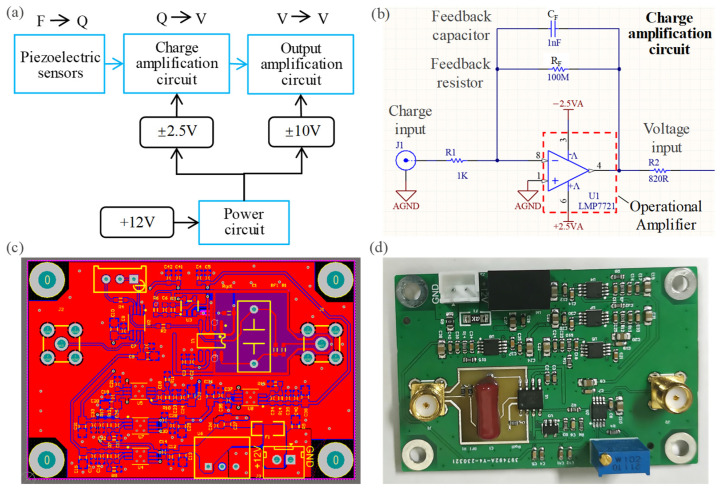
Homemade charge amplifier: (**a**) illustration; (**b**) charge amplification circuit; (**c**) design drawing of the charge amplifier; (**d**) physical photograph of the charge amplifier.

When a piezoelectric force sensor is subjected to a force, it generates an electric charge. The charge must be amplified and converted into an analog voltage signal suitable for data acquisition by a charge amplifier so that the magnitude of the applied force can be calculated. In this paper, a homemade charge amplifier was designed, which mainly consists of a charge amplifier circuit, an output amplifier circuit, and a power circuit, as shown in Figure 4a. The charge amplification circuit is the core part of the charge amplifier, and its schematic diagram is illustrated in Figure 4b. It is composed of a high-gain operational amplifier, a feedback capacitor *C_F_* and a feedback resistor *R_F_*. The feedback capacitor stores the charge generated by the piezoelectric sensor, while the feedback resistor and the feedback capacitor form a discharge loop to reset the output voltage to zero. The input bias current of the operational amplifier in the charge amplification circuit directly affects the measurement accuracy, so an operational amplifier with ultra-low bias current is required. The operational amplifier used in this work is the LMP7721, which features an extremely low bias current of only 3 fA at minimum and a maximum input bias current of ±25 fA at 25 °C. This operational amplifier is powered by ±2.5 V. The output amplifier circuit is constructed using a low-power and high-speed instrumentation amplifier AD8421, which provides a ±10 V output voltage with a gain of 5. To meet the power supply demands of all circuits, a power circuit was designed, which is powered by a single 12 V lithium-ion battery and converts the required voltages for each module. In practical applications, the noise level of the charge amplifier output signal is highly sensitive to power supply ripple. Low-dropout linear regulators are a common solution to suppress power supply ripple. In this design, TPS7A4901 and TPS7A3001 are selected as the voltage regulator chips, which feature low noise (15.40 μVRMS) and can deliver a supply current of 150 mA. Figure 4c and Figure 4d show the design drawing and the physical photograph of the charge amplifier, respectively.

### 3.2. Three-Axis Force Calibration Test

**Figure 5:**
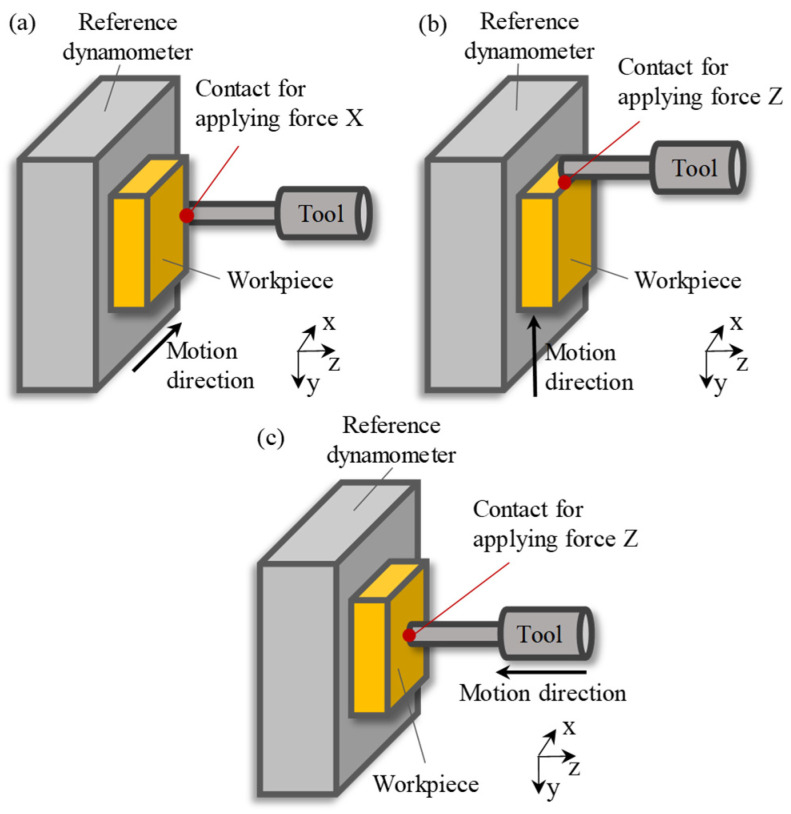
Three-axis force calibration method: (**a**) X; (**b**) Y; (**c**) Z.

The three-axis force calibration method involves driving the machine tool to make the workpiece on the dynamometer contact the diamond milling tool to generate an interaction force, as shown in Figure 5. Therefore, the force loaded on the workpiece side is the same as that on the tool. The output of the standard dynamometer is recorded as the calibration reference value. In order to make the static calibration process as consistent as possible with the cutting process, the protruding length of the tool is kept the same as that in the ultra-precision milling experiments to reduce calibration errors.

In this way, multiple different forces were generated along each axial direction. Figure 5 shows the results of the outputs of the sensors under different applied forces. As can be seen in the figure, the outputs of all the sensors exhibit a good linear relationship with the applied forces. There is a certain degree of variation in the output sensitivity of each sensor, which is mainly attributable to factors such as inconsistent preload forces, positional symmetry errors in mounting, and anisotropic stiffness of the milling spindle.

**Figure 6:**
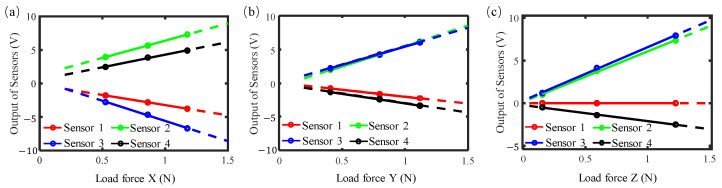
Outputs of the sensors under different applied forces: (**a**) load in X direction; (**b**) load in Y direction; (**c**) load in Z direction.

Based on the calibration results in Figure 6, a transformation matrix from forces to outputs of sensors can be obtained by:

(4) $$[U_{1}\;U_{2}\;U_{3}\;U_{4}{]}^{T}=K^{+}\cdot \;[F_{x}\;F_{y}\;F_{z}{]}^{T}$$

Then, the transformation matrix from outputs of sensors to forces can be calculated by:

(5) $$K={(K}^{{+}^{T}}K{)}^{-1}\cdot K^{{+}^{T}}=\left[\begin{array}{c} -0.0045\;\;\;0.0793\;-0.0553\;\;\;0.0653\\ -0.3445\;-0.0490\;-0.0327\;-0.2631\\ \;0.3307\;\;\;0.1378\;\;\;0.1019\;\;\;0.2410 \end{array}\right]\;$$

**Figure 7:**
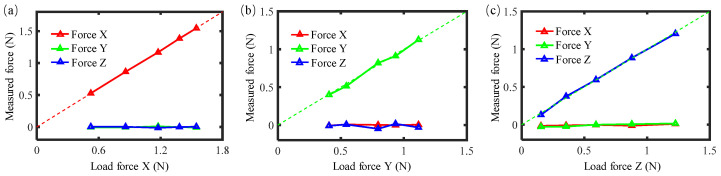
The corrected calibration results: (**a**) load in X direction; (**b**) load in Y direction; (**c**) load in Z direction.

To verify the accuracy of the calibration, tests were conducted by generating multiple forces between the tool and the workpiece along the X-, Y-, and Z-directions respectively. Based on Equation (3), the three-axis forces derived from the raw output signals of the piezoelectric sensors were calculated, and the corrected calibration results are presented in Figure 7. As shown in the figure, the application of the linear transformation matrix can effectively correct the measured forces, with the force measurement linearity in all three directions being within 2%.

### 3.3. Tool-Setting Test

**Figure 8:**
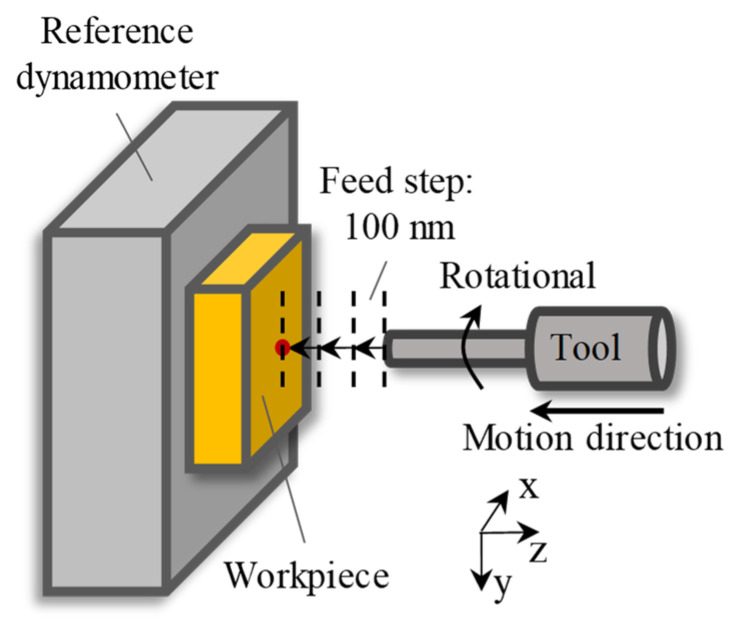
Method of tool-setting test.

**Figure 9:**
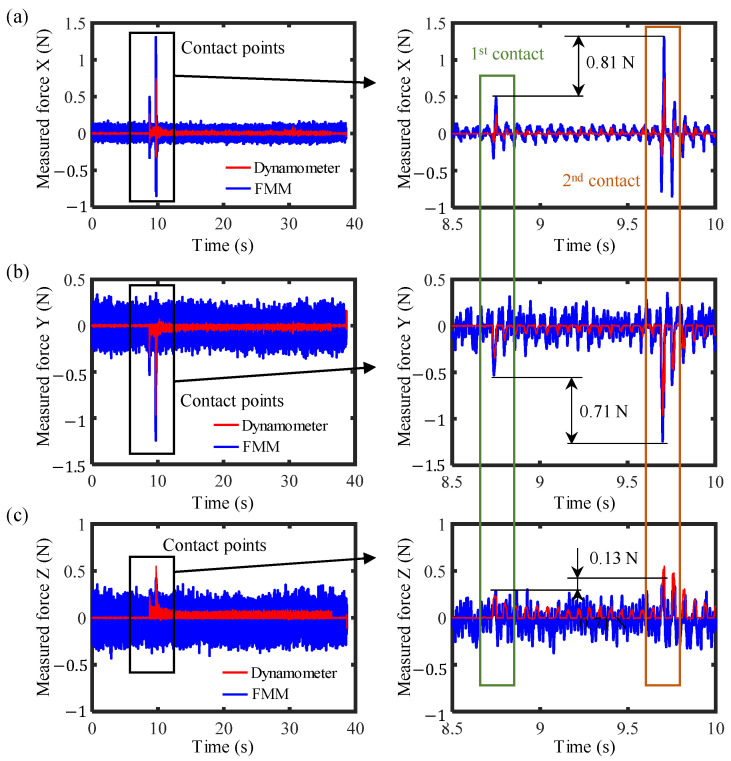
The measured three-axis forces in the tool-setting process: (**a**) measured force in X direction; (**b**) measured force in Y direction; (**c**) measured force in Z direction.

Force measurement resolution is a critical parameter for the ultra-precision milling process; the smaller the force measurement resolution, the better it can enable the monitoring of a smaller material removal process. Dynamic tool-setting experiments were conducted to test the level of force measurement resolution during the milling process. Figure 8 shows the schematic of the tool-setting test. In the tool-setting test, the tool needs to rotate to generate cutting force when the tool contacts the workpiece. The rotational speed of the milling spindle was set to a commonly used value of 1000 rpm, and the tool was driven close to the workpiece until contact with a feed step of 100 nm. The workpiece material of this experiment was copper. The measured three-axis forces by the force measurement mechanism (FMM in the figure) and the dynamometer in the tool-setting process are illustrated in Figure 9. The first contact between the tool and the workpiece should occur with a material removal depth of less than 100 nm, and the second contact with that of 100 nm. As can be seen from the figure, the force measurement mechanism can clearly identify the force caused by a material removal depth of 100 nm, with the variations in forces being 0.81 N, 0.71 N and 0.13 N in the X-, Y-, and Z-directions, respectively. Based on the results, the radial force is much greater than the axial force during this process. This result demonstrated the capacity of the force measurement mechanism for tool-setting accuracy of less than 100 nm.

## 4. Cutting Experiment and Discussion

**Figure 10:**
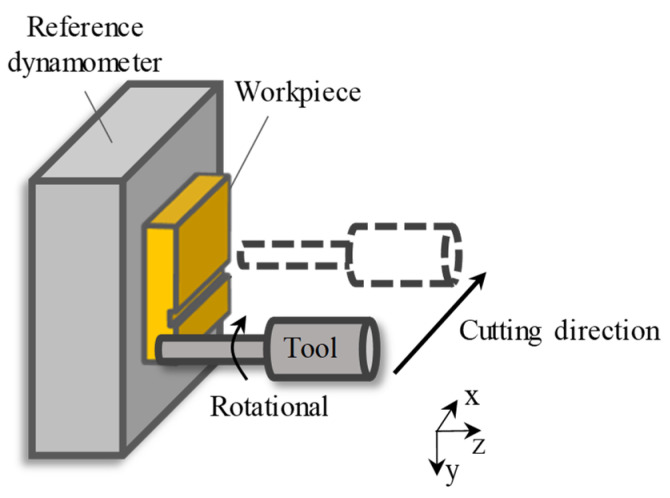
Schematic of the cutting experiment.

Ultra-precision milling experiments of grooves with constant depth were conducted to verify the capacity of the in-process force measurement of the proposed method. Figure 10 shows the schematic of the milling experiment. The spindle speed was set to 2000 rpm, the axial cutting depth was 1 μm, the feed direction was set to be along the X-direction, the feed rate was 6 mm/min, and the workpiece material was copper. The cutting parameters above are common parameters in actual milling processes.

**Figure 11:**
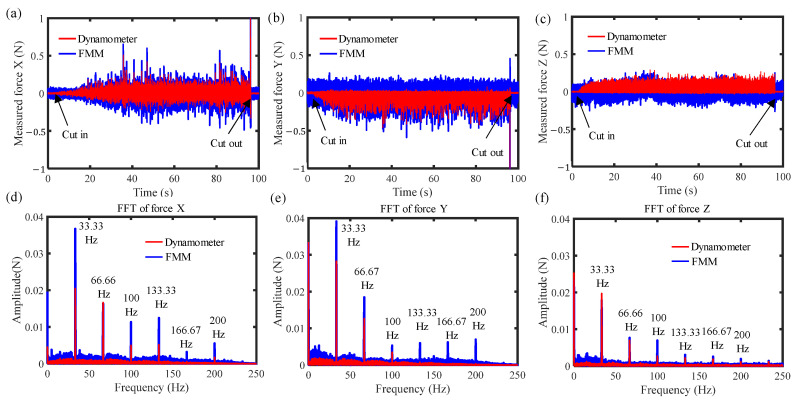
The measured three-axis forces in cutting process: (**a**) measured force in X direction; (**b**) measured force in Y direction; (**c**) measured force in Z direction; (**d**) FFT of force in X direction; (**e**) FFT of force in Y direction; (**f**) FFT of force in Z direction.

During the milling process, the dynamometer and the developed force measurement mechanism (FMM in the figure) measured cutting forces simultaneously, and the measurement results of the three-axis forces are shown in Figure 11. For the time-domain measurement results in the X- and Y-directions, as illustrated in Figure 11a,b, it can be observed that the waveform of the measurement results obtained by the developed force measurement mechanism is approximately consistent with that of the reference dynamometer. In addition, the cutting force in the X-direction exhibits both positive and negative values, while the force in the Y-direction shows only negative values, which is consistent with the force characteristics of milling process with feeding along the X-direction. As for the time-domain measurement results in the Z-direction, shown in Figure 11c, the cutting force is much smaller than that in X- and Y-directions, and the measured forces from the force measurement mechanism is close to the noise level itself. Figure 11d–f show the frequency-domain comparison of the measured forces. As can be observed from Figure 11d,e, the measured forces from the force measurement mechanism have larger amplitudes at the spindle rotation frequency (33.33 Hz) and other non-characteristic frequencies, which are caused by inertial forces and noise.

To ensure the accuracy of the force measurement results, a Wiener filter was used to eliminate the effects of inertial forces and noise [30]. The Wiener filter is based on the minimum mean square error criterion. After a noisy original signal passes through a Wiener filter, the output signal is a minimum mean square error estimate of the noise-free actual signal, thus achieving the effect of noise reduction. The expression of the Wiener filter in the frequency-domain is as follows:

(6) $$\tilde{D}\left(\omega_{k}\right)=H\left(\omega_{k}\right)Y(\omega_{k})$$

where $\tilde{D}\left(\omega_{k}\right)$ is the output signal from the filter, $H\left(\omega_{k}\right)$ is the filter, $Y(\omega_{k})$ is the original signal. The error $E\left(\omega_{k}\right)$ between the output signal from the filter $\tilde{D}\left(\omega_{k}\right)$ and the actual free-noise signal $D\left(\omega_{k}\right)$ can be expressed as:

(7) $$E\left(\omega_{k}\right)=D\left(\omega_{k}\right)-\tilde{D}\left(\omega_{k}\right)=D\left(\omega_{k}\right)-H\left(\omega_{k}\right)Y(\omega_{k})$$

To achieve the minimum mean square error estimation of the error, the partial derivative of the mean square error function with respect to the filter should be zero:

(8) $$\frac{\partial E\left[{\left|E\left(\omega_{k}\right)\right|}^{2}\right]}{\partial H\left(\omega_{k}\right)}=H\left(\omega_{k}\right)P_{yy}\left(\omega_{k}\right)-P_{yd}\left(\omega_{k}\right)=0$$

where $P_{yy}\left(\omega_{k}\right)$ is the auto-power spectrum of the original signal, $P_{yd}\left(\omega_{k}\right)$ is the cross-power spectrum between the original signal and the actual free-noise signal. Based on Equation (8), the filter can be expressed as:

(9) $$H\left(\omega_{k}\right)=\frac{P_{dy}\left(\omega_{k}\right)}{P_{yy}\left(\omega_{k}\right)}$$

The original signal $Y\left(\omega_{k}\right)$ is the sum of the actual free-noise signal $D\left(\omega_{k}\right)$ and the noise signal $N(\omega_{k})$:

(10) $$Y\left(\omega_{k}\right)=D\left(\omega_{k}\right)+N(\omega_{k})$$

Combining Equations (8) and (9), the filter can be expressed as:

(11) $$H\left(\omega_{k}\right)=\frac{P_{yy}\left(\omega_{k}\right)-P_{nn}\left(\omega_{k}\right)}{P_{yy}\left(\omega_{k}\right)}$$

where $P_{nn}\left(\omega_{k}\right)$ is the auto-power spectrum of the noise signal.

**Figure 12:**
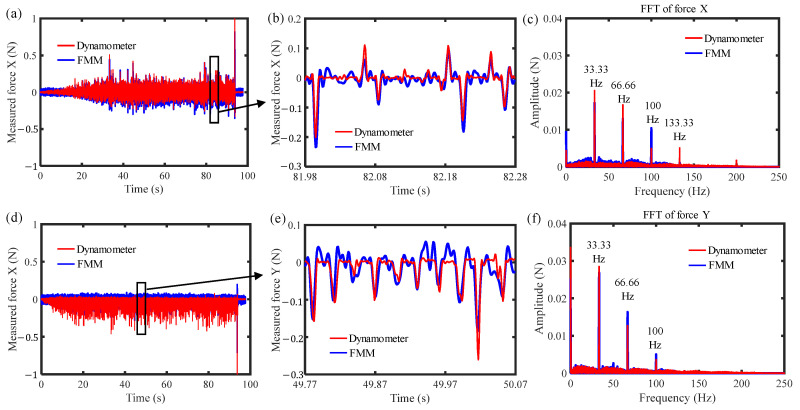
The filtered forces: (**a**) filtered force in X direction; (**b**) local force in X direction; (**c**) FFT of force in X direction; (**d**) filtered force in Y direction; (**e**) local force in Y direction; (**f**) FFT of force in Y direction.

The noise signals were obtained by collecting the measured forces while conducting the same motion path of the machine but without cutting between the tool and the workpiece. Then, based on Equation (11), the filtered forces were obtained. Figure 12 shows the filtered forces in the X- and Y-directions. As can be seen in Figure 12a,d, the variations in the measured forces after filtering are more consistent with those measured by the dynamometer. Figure 12b,e show the comparisons of local forces, which further demonstrate that the measurement results are basically consistent with the waveform variations and amplitude magnitudes of the dynamometer. The minimum identifiable characteristic cutting force is below 50 mN, which verifies the effectiveness of the force measurement mechanism in detecting micro-cutting forces during the ultra-precision milling process. Meanwhile, Figure 12c,f present the frequency-domain comparison of the filtered force signals in the X- and Y-directions. As can be seen in the figures, after filtering, the amplitude of the force signals at the spindle rotation frequency (33.33 Hz) is significantly reduced, and the amplitudes at all non-characteristic frequencies are also decreased, making the amplitudes much closer to those measured by the dynamometer. Since the cutting forces are mainly concentrated at the spindle rotation frequency, which is consistent with the milling process, this also verifies the effectiveness of the force measurement mechanism in detecting cutting forces in ultra-precision milling.

To further evaluate the measurement accuracy of the force measurement mechanism, the cross-correlation coefficient between the forces measured by the force measurement mechanism and the reference dynamometer was calculated by:

(12) $$r=\frac{Cov(F_{f},\;F_{d})}{\sqrt{Var\left[F_{f}\right]Var[F_{d}]}}=\frac{\sum_{i=1}^{N}\left(F_{f}\left(i\right)-\bar{F_{f}})(F_{d}\left(i\right)-\bar{F_{d}}\right)}{\sqrt{\sum_{i=1}^{N}(F_{f}\left(i\right)-\bar{F_{f}}{)}^{2}\sum_{i=1}^{N}(F_{d}\left(i\right)-\bar{F_{d}}{)}^{2}}}$$

where *Cov* denotes covariance, *Var* denotes variance, and *F_f_* and *F_d_* represent the force signals measured from the developed force measurement mechanism and the dynamometer, respectively. Based on Equation (11), the correlation coefficients of the measured forces in the X- and Y-directions can be calculated as 0.84 and 0.77, respectively, demonstrating a high correlation between the force measurement mechanism and the reference dynamometer. The discrepancy between these two signals is mainly attributable to the differences in force transmission paths and residual noise signals.

The aforementioned ultra-precision milling experiments have verified the millinewton-level three-axis precision cutting force measurement capability of the developed force measurement mechanism with a relatively low material removal rate, providing a promising technical approach for ultra-precision milling process monitoring and process optimization.

## 5. Conclusions

This paper proposes a piezoelectric spindle-integrated three-axis cutting force measurement system with both high-stiffness and high-sensitivity for ultra-precision diamond milling. The primary contributions and conclusions of this work are as follows:

(1) Considering both high-stiffness and high-sensitivity, a spindle-integrated force measurement mechanism was designed and integrated on an ultra-precision machine tool, which employs four groups of piezoelectric sensors arranged symmetrically and diagonally to measure three-axis cutting forces.

(2) Calibration tests were conducted, and the results showed good linearity of force detection in the three axial directions with an error of less than 2%.

(3) A tool-setting experiment based on force detection signals was conducted, verifying its Z-direction precision tool-setting capability with an accuracy of less than 100 nm, which exhibits high accuracy for obtaining the cutting origin.

(4) Ultra-precision milling experiments were carried out, and the measurement noise from vibration and inertial forces under spindle rotation was considered and filtered for accurate force measurement. The measurement results indicate that the developed force measurement mechanism can effectively identify three-axis cutting forces below 50 mN, and its measurement results show good correlation with those obtained from the standard dynamometer.

This paper provides an effective approach for precision tool-setting, identification of abnormal conditions such as micro tool wear and slight chatter, and process optimization, based on precision in-process force measurement in ultra-precision milling processes. The current limitations include unavoidable measurement error of static force due to the charge leakage of piezoelectric sensors, and a time-consuming assembly process. Future work will focus on artificial intelligence algorithm for eliminating measurement error of static force, sensitivity enhancement, prediction of machined surface topography and roughness, and other related aspects.

## Data Availability

Data underlying the results presented in this paper are available from the authors upon request.

## References

[1] Fang F., Lai M., Wang J., Luo X., Yan J., Yan Y. (2022). Nanometric cutting: Mechanisms, practices and future perspectives. Int. J. Mach. Tools Manuf..

[2] Zhang S., Zhou Y., Zhang H., Xiong Z., To S. (2019). Advances in ultra-precision machining of micro-structured functional surfaces and their typical applications. Int. J. Mach. Tools Manuf..

[3] Ibaraki S., Shimizu T. (2010). A long-term control scheme of cutting forces to regulate tool life in end milling processes. Precis. Eng..

[4] Bo Q., Wang P., Hou B., Liu H., Li X., Li T., Wang Y. (2024). Mirror supporting device based on magnetorheological fluid and control strategy based on force signal feedback for mirror milling. Mech. Syst. Signal Process..

[5] Sugita N., Nakano T., Nakajima Y., Fujiwara K., Abe N., Ozaki T., Suzuki M., Mitsuishi M. (2009). Dynamic controlled milling process for bone machining. J. Mater. Process. Technol..

[6] Deng Z., Jin H., Hu Y., He Y., Zhang P., Tian W., Zhang J. (2016). Fuzzy force control and state detection in vertebral lamina milling. Mechatronics.

[7] Li Z., Chen Y.L. (2024). Controllable diamond cutting of structured surfaces with subnanometric height features on silicon. Precis. Eng..

[8] Schmucker B., Wang C.P., Zaeh M.F., Erkorkmaz K. (2023). Wide-bandwidth cutting force monitoring via motor current and accelerometer signals. CIRP Ann..

[9] Li X., Venuvinod P.K., Chen M.K. (2000). Feed cutting force estimation from the current measurement with hybrid learning. Int. J. Adv. Manuf. Technol..

[10] Qin Y., Wang D., Yang Y. (2020). Integrated cutting force measurement system based on MEMS sensor for monitoring milling process. Microsyst. Technol..

[11] Ting Y., Chen H.Y., Chen J.H., Suprapto, Yu C.-H. (2021). Design and performance evaluation of a multi-axis thin-film sensor for milling process measurement. Sens. Actuators A Phys..

[12] Chae J., Park S.S. (2007). High frequency bandwidth measurements of micro cutting forces. Int. J. Mach. Tools Manuf..

[13] Gomi N., Ishii N., Hozumi R., Kumehara H. (2011). End-Mill Evaluation by Measurement of Cutting Force. Mater. Sci. Forum.

[14] Xie Z., Lu Y., Li J. (2017). Development and testing of an integrated smart tool holder for four-component cutting force measurement. Mech. Syst. Signal Process..

[15] Rizal M., Ghani J.A., Nuawi M.Z., Haron C.H.C. (2018). An embedded multi-sensor system on the rotating dynamometer for real-time condition monitoring in milling. Int. J. Adv. Manuf. Technol..

[16] Xie Z., Li J., Lu Y. (2018). An integrated wireless vibration sensing tool holder for milling tool condition monitoring. Int. J. Adv. Manuf. Technol..

[17] Liu M., Bing J., Xiao L., Yun K., Wan L. (2018). Development and testing of an integrated rotating dynamometer based on fiber Bragg grating for four-component cutting force measurement. Sensors.

[18] Qin Y., Zhao Y., Li Y., Zhao Y., Wang P. (2017). A novel dynamometer for monitoring milling process. Int. J. Adv. Manuf. Technol..

[19] Zhang P., Gao D., Lu Y., Wang F., Liao Z. (2022). A novel smart toolholder with embedded force sensors for milling operations. Mech. Syst. Signal Process..

[20] Luo M., Luo H., Axinte D., Liu D., Mei J., Liao Z. (2018). A wireless instrumented milling cutter system with embedded PVDF sensors. Mech. Syst. Signal Process..

[21] Yau H.T., Hong S.W., Sue C.Y., Tsao T.-C. (2024). Novel Sensory Tool Holder Design and Optimization for Multiaxis Cutting Force Sensing in Manufacturing. IEEE/ASME Trans. Mechatron..

[22] Albrecht A., Park S.S., Altintas Y., Pritschow G. (2005). High frequency bandwidth cutting force measurement in milling using capacitance displacement sensors. Int. J. Mach. Tools Manuf..

[23] Kim J.H., Chang H.K., Han D.C., Jang D.Y., Oh S.I. (2005). Cutting force estimation by measuring spindle displacement in milling process. CIRP Ann..

[24] Sarhan A.A.D., Matsubara A., Sugihara M., Saraie H., Ibaraki S., Kakino Y. (2006). Monitoring method of cutting force by using additional spindle sensors. JSME Int. J. Ser. C Mech. Syst. Mach. Elem. Manuf..

[25] Denkena B., Boujnah H. (2018). Feeling machines for online detection and compensation of tool deflection in milling. CIRP Ann..

[26] Boujnah H., Irino N., Imabeppu Y., Kawai K., Mori M. (2022). Spindle-integrated, sensor-based measurement system for cutting forces. CIRP Ann..

[27] Gao W., Chen Y.L., Lee K.W., Noh Y.-J., Shimizu Y., Ito S. (2013). Precision tool setting for fabrication of a microstructure array. CIRP Ann..

[28] Chen Y.L., Cai Y., Tohyama K., Shimizu Y., Ito S., Gao W. (2017). Auto-tracking single point diamond cutting on non-planar brittle material substrates by a high-rigidity force controlled fast tool servo. Precis. Eng..

[29] Li Z., An L., Lin H., Chen Y.-L. (2025). Development of an optimized smart tool holder using symmetrical structure for three-axis cutting force measurement in diamond cutting. Precis. Eng..

[30] Nagayama K. (2025). Wiener filter unifies Hilbert and Zernike phase plates in electron microscopy. Biophys. Rev..

